# A Fast Method for the Segmentation of Synaptic Junctions and Mitochondria in Serial Electron Microscopic Images of the Brain

**DOI:** 10.1007/s12021-015-9288-z

**Published:** 2016-01-16

**Authors:** Pablo Márquez Neila, Luis Baumela, Juncal González-Soriano, Jose-Rodrigo Rodríguez, Javier DeFelipe, Ángel Merchán-Pérez

**Affiliations:** Departamento de Inteligencia Artificial, Universidad Politécnica de Madrid, 28660 Boadilla del Monte, Madrid, Spain; Departamento de Anatomía. Facultad de Veterinaria, Universidad Complutense, Madrid, Spain; Laboratorio Cajal de Circuitos Corticales, Centro de Tecnología Biomédica, Universidad Politécnica de Madrid and Instituto Cajal, CSIC, Madrid, Spain; Departamento de Arquitectura y Tecnología de Sistemas Informáticos, Facultad de Informática, Universidad Politécnica de Madrid, Madrid, Spain

**Keywords:** Three-dimensional electron microscopy, Automatic image segmentation, cerebral cortex, Mitochondria, Synapses

## Abstract

Recent electron microscopy (EM) imaging techniques permit the automatic acquisition of a large number of serial sections from brain samples. Manual segmentation of these images is tedious, time-consuming and requires a high degree of user expertise. Therefore, there is considerable interest in developing automatic segmentation methods. However, currently available methods are computationally demanding in terms of computer time and memory usage, and to work properly many of them require image stacks to be isotropic, that is, voxels must have the same size in the X, Y and Z axes. We present a method that works with anisotropic voxels and that is computationally efficient allowing the segmentation of large image stacks. Our approach involves anisotropy-aware regularization via conditional random field inference and surface smoothing techniques to improve the segmentation and visualization. We have focused on the segmentation of mitochondria and synaptic junctions in EM stacks from the cerebral cortex, and have compared the results to those obtained by other methods. Our method is faster than other methods with similar segmentation results. Our image regularization procedure introduces high-level knowledge about the structure of labels. We have also reduced memory requirements with the introduction of energy optimization in overlapping partitions, which permits the regularization of very large image stacks. Finally, the surface smoothing step improves the appearance of three-dimensional renderings of the segmented volumes.

**Electronic supplementary material** The online version of this article (doi:10.1007/s12021-015-9288-z) contains supplementary material, which is available to authorized users.

## Introduction

The availability of technologies such as combined Focused Ion Beam milling/Scanning Electron Microscopy (FIB/SEM) and Serial Block-Face Scanning Electron Microscopy (SBFSEM) for the study of biological tissues permits the automated acquisition of large numbers of serial sections from brain samples (see for example (Denk and Horstmann 2004; Knott et al. 2008; Merchan-Perez et al. 2009)). These three-dimensional samples contain invaluable structural information that must be extracted from the stack of serial images. Electron micrographs of nervous tissue typically show a large variety of structures, such as neuronal and glial processes with their corresponding cytoplasmic organelles (e.g., vesicles, tubules, filaments and mitochondria) and synapses. From a practical point of view, manual segmentation of these structures is a difficult and time-consuming task that requires a high degree of expertise. As a consequence, much effort has been devoted to the development of automated algorithms.

Brain images produced by electron microscopy (EM) are very complex and noisy with strong gray-level gradients that do not always correspond to region boundaries. Moreover, different neuronal structures may have similar local image appearance. Hence, it is extremely difficult to develop a fully automated segmentation algorithm. Although automated image processing techniques have addressed the problem of membrane detection and dendrite reconstruction (Turaga et al. 2010), standard computer vision algorithms used for the segmentation of textures (Haindl and Mikes 2008) or natural images (Martin et al. 2001) perform poorly, and standard techniques for the segmentation of biomedical images such as contour evolution (Jurrus et al. 2009) cannot handle the abundant image gradients.

Among the various structures visualized with EM, mitochondria and the synaptic junctions are of particular interest to neuroscience. Indeed, most information in the mammalian nervous system flows though chemical synapses. Thus, the quantification and measurement of synapses is a major goal in the study of brain synaptic organization in both health and disease (DeFelipe 2010). Mitochondria are organelles that produce most of the cell’s supply of adenosine triphosphate (ATP) which transports chemical energy within cells for metabolism. In addition to supplying cellular energy, mitochondria are involved in many other crucial cellular physiological tasks (*e.g.*, McBride et al. 2006) and their alterations have been associated with a number of diseases such as Alzheimer’s disease (*e.g.*, Santos et al. 2010). Therefore, substantial effort has been put into developing methods for accurate segmentation of synapses and mitochondria in the brain.

Although there are good practical synapse segmentation approaches relying on semi-automated tools (Morales et al. 2011), recent research has focused on machine learning approaches to diminish the degree of user interaction. (Becker et al. 2013) introduced a synaptic junction segmentation approach specifically designed for isotropic resolution image stacks, that is, stacks where voxel dimensions were identical in all X, Y and Z-axes. This method is based on a boosting algorithm that discovers local context cues related to the presence of the synaptic junction. The local context around potential synapse-like regions is also used in (Jagadeesh et al. 2013). However, the approach of Jagadeesh et al. relies on a computationally demanding set of image features that require up to 12 hours of computing time in a 32-node cluster. An alternative way of detecting synapses is by selectively staining them with ethanolic phosphotungstic acid (Navlakha et al. 2013), although this obscures other subcellular details and the tissue preservation is not appropriate for detailed ultrastructural analysis. Finally, (Kreshuk et al. 2011) used the Ilastik toolbox to segment synaptic junctions.

Several algorithms have been specifically designed to segment mitochondria in EM images. A texton-based approach comparing K-NN, SVM and AdaBoost classifiers was proposed (Narasimha et al. 2009). Lucchi and colleagues (Lucchi et al. 2012) later introduced an algorithm using as input 3D supervoxels and assuming almost isotropic image stacks. A different approach has been presented by Giuly and colleagues (Giuly et al. 2012). Their method performs the segmentation of mitochondria in anisotropic stacks of images. However, it is computationally very expensive and requires long processing times.

Consequently, our aim was to develop a method that does not require isotropic voxels and that is computationally efficient to allow the interactive segmentation of large image stacks that are now available. Moreover, our approach also involves image regularization and surface smoothing techniques to improve the segmentation.

## Material & Methods

For the development of our segmentation algorithm we have used FIB/SEM image stacks that have been acquired in our laboratory from the rat somatosensory cortex (Merchan-Perez et al. 2009; Anton-Sanchez et al. 2014). The resolution was always the same in the X and Y axes and ranged from 3.7 nm per pixel to 14.7 nm per pixel. Resolution in the Z axis, equivalent to section thickness, was in all cases 20 nm per pixel. The stacks were thus anisotropic, that is, they did not have the same resolution in all three axes. Our segmentation algorithm has been specifically designed to take anisotropy into account, and we define the anisotropy factor as:

1 $$ \rho = \frac{\textrm{Voxel size in the Z~axis}}{\textrm{Voxel size in the X (or Y) axis}} $$

Thus, our stacks had anisotropy factors ranging from 5.41 to 1.36. To make our method comparable to others, we used an additional stack of SBFSEM images available online with an anisotropy factor *ρ* = 5.

### Description of the Segmentation Algorithm

**Fig. 1:**
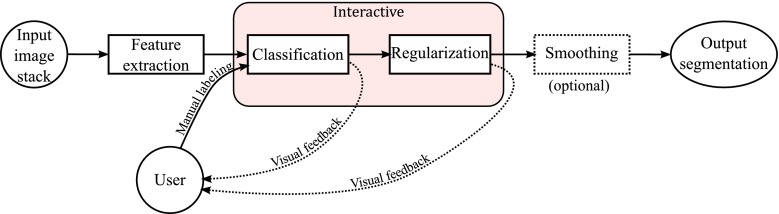
Workflow of the segmentation algorithm. The input stack of serial images is subjected to four successive steps, two of which can be interactively modified by the user

Our algorithm learns to detect and segment potentially any type of structure from the visual information in a stack of FIB/SEM or SBFSEM images, although in this work we have focused on synaptic junctions and mitochondria. To this end, the algorithm must be trained by providing samples of segmented structures. The user provides these samples in an interactive way using an in-house application we have developed: first, a few voxels are manually labeled as mitochondria, for example, using standard tools such as the two or three-dimensional brush. The system then performs an automatic segmentation based on the training given, thus providing visual feedback. The user then refines the training by labeling new samples in the areas where the automatic segmentation is wrong. This procedure is repeated until the results are satisfactory (see Fig. 1).

Our automatic segmentation algorithm has three steps: feature extraction, voxel-wise classification and regularization. An optional fourth step, smoothing, enhances the visual appearance of the segmentation when it is rendered in 3D.

#### Feature Extraction

Feature extraction is performed on all voxels in the stack. The features of a voxel are a vector of real numbers that concisely describe the relevant visual information in the vicinity of that voxel. A feature extractor is a function from the space of EM stacks to the space of feature stacks. We have developed two feature extractors, F2D and F3D, which aggregate visual information around each voxel at several scales, and are rotationally invariant and robust to the noise present in EM images. F3D is a feature extractor that takes into account three-dimensional neighborhoods around each voxel. It is adequate for isotropic stacks. F2D, on the other hand, extracts a feature vector for each pixel in an image of the stack considering visual information of a neighborhood of the pixel in that slice and ignoring the information in other slices. F2D is a feature extractor that is suitable for anisotropic stacks. In the paragraphs that follow, we first describe F2D and then introduce F3D as a generalization.

F2D works on each image of the stack separately. Hence we only consider a single image *I* in the following description. F2D first applies a set of linear operators (zero, first and second order derivatives) to the smoothed image I at several scales. Thus, the set of linear operators at a scale *σ* is

2 $$\begin{array}{@{}rcl@{}} \left\{G_{\sigma}*, \sigma\cdot G_{\sigma}*\frac{\partial}{\partial x}, \sigma\cdot G_{\sigma}*\frac{\partial}{\partial y}, \sigma^{2}\cdot G_{\sigma}*\frac{\partial^{2}}{\partial x^{2}}, \sigma^{2}\right.\\[-2pt] \left.\cdot ~G_{\sigma}*\frac{\partial^{2}}{\partial xy}, \sigma^{2}\cdot G_{\sigma}*\frac{\partial^{2}}{\partial y^{2}} \right\}, \end{array} $$

where *G*_*σ*_ is a Gaussian filter of radius *σ* and ∗ is the convolution operator. The response to these operators will be noted as *s*_00_, *s*_10_, *s*_01_, *s*_20_, *s*_11_ and *s*_02_ (note that the subscripts denote the order of the derivatives). With these responses to the filters at a scale *σ*, we can compute a partial feature vector for the pixels of *I* at that scale. This partial feature vector has four components:

3 $$ \left\{s_{00}, \sqrt{s_{10}^{2}+s_{01}^{2}}, \lambda_{1}, \lambda_{2}\right\}, $$

where *s*_00_ is the smoothed image, $\sqrt {s_{10}^{2}+s_{01}^{2}}$ is the gradient magnitude and *λ*_1_ and *λ*_2_ are the first and second eigenvalues of the Hessian matrix, that are computed as

4 $$\begin{array}{@{}rcl@{}} \lambda_{1} &=& \frac{1}{2}\left( s_{20} + s_{02} + \sqrt{\left( s_{20} + s_{02}\right)^{2} + 4s_{11}^{2}}\right), \end{array} $$

5 $$\begin{array}{@{}rcl@{}} \lambda_{2} &=& \frac{1}{2}\left( s_{20} + s_{02} - \sqrt{\left( s_{20} + s_{02}\right)^{2} + 4s_{11}^{2}}\right). \end{array} $$

**Fig. 2:**
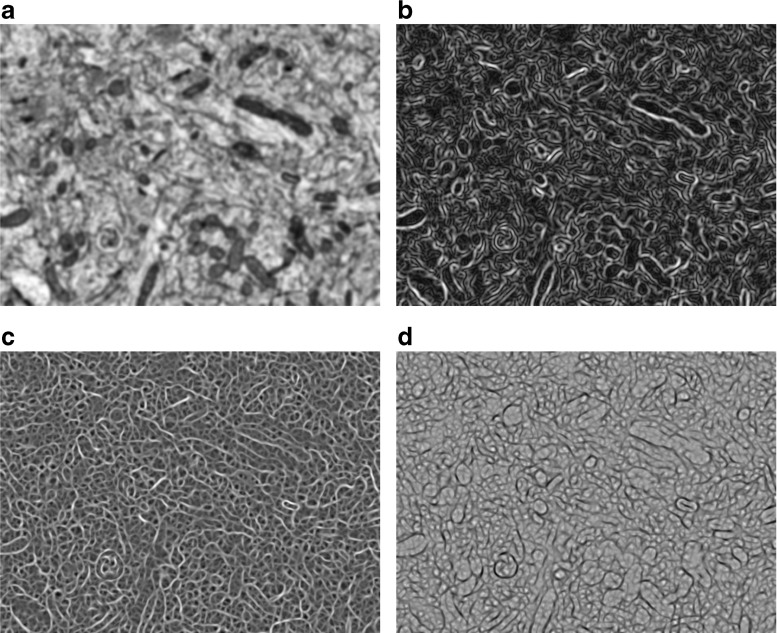
Feature extraction from a single image. In this example, feature extraction has been performed at a scale *σ* = 4 pixels. The resulting partial feature vector has four components: the smoothed image (**a**), the gradient magnitude (**b**), and the first (**c**) and second eigenvalues (**d**) of the Hessian matrices. Feature extraction is also performed at several other scales (not shown) to obtain the complete feature vector

Figure 2 shows the components of a partial feature vector at a fixed scale for all the pixels in a single image.

The complete feature vector for each pixel is the concatenation of several partial feature vectors. We apply this procedure at *n* different scales {*σ*_0_,…, *σ*_*n*−1_}, producing a feature vector with 4*n* components for each pixel in *I*. The set of scales should match the size of the structures that have to be detected in the images. In practice, the user only sets the initial scale *σ*_0_, which we call the base scale, and the rest of scales are given by $\sigma _{i} = 2^{\frac {1}{2}i}\sigma _{0}$. For example, if we use *n* = 4 scales and set the smallest scale to *σ*_0_ = 4 pixels, our feature vectors will have 16 dimensions and they will range from 4 to 11.31 pixels in scale.

F3D is a generalization of F2D for isotropic image stacks. As in F2D, the set of linear operators with the zero, first and second order derivatives at a given scale *σ*,

6 $$ \left\{ \begin{array}{l} G_{\sigma}*, \sigma\cdot G_{\sigma}*\frac{\partial}{\partial x}, \sigma\cdot G_{\sigma}*\frac{\partial}{\partial y}, \sigma\cdot G_{\sigma}*\frac{\partial}{\partial z}, \\ \sigma^{2}\cdot G_{\sigma}*\frac{\partial^{2}}{\partial x^{2}}, \sigma^{2}\cdot G_{\sigma}*\frac{\partial^{2}}{\partial xy}, \sigma^{2}\cdot G_{\sigma}*\frac{\partial^{2}}{\partial xz}, \\ \sigma^{2}\cdot G_{\sigma}*\frac{\partial^{2}}{\partial y^{2}}, \sigma^{2}\cdot G_{\sigma}*\frac{\partial^{2}}{\partial yz}, \sigma^{2}\cdot G_{\sigma}*\frac{\partial^{2}}{\partial z^{2}} \\ \end{array} \right\}, $$

is applied to the stack obtaining the responses {*s*_*ijk*_:*i* + *j* + *k* ≤ 2}, where *i*, *j* and *k* indicate the order of the derivatives in the X, Y and Z axes. The partial feature vector for each voxel of the stack at a given scale is

7 $$ \left\{s_{000}, \sqrt{s_{100}^{2} + s_{010}^{2} + s_{001}^{2}}, \lambda_{1}, \lambda_{2}, \lambda_{3}\right\}, $$

where the first component is the smoothed image, the second one is the magnitude of the gradient, and *λ*_1_, *λ*_2_ and *λ*_3_ are the eigenvalues of the Hessian matrix

8 $$ \begin{pmatrix} s_{200} & s_{110} & s_{101} \\ s_{110} & s_{020} & s_{011} \\ s_{101} & s_{011} & s_{002} \end{pmatrix}. $$

Again, the complete feature vector for each voxel is the concatenation of the partial feature vectors at several scales.

#### Classification

A classifier uses the feature vectors to determine the probability that a voxel belongs to each label. This classifier has to be trained with labeled data to learn the relationship between feature vectors and labels. Here we briefly present how the classifier is trained and how a trained classifier can be used with new unclassified data.

Our classifier learns the probability distribution *P*(*y*_*i*_∣*f*_*i*_(**x**)) of the label *y*_*i*_ for the pixel *i* given the observed feature vector *f*_*i*_(**x**). We use the Bayes’ rule to express this distribution as a product

9 $$ P(y_{i}\mid f_{i}(x)) \propto p(f_{i}(\mathbf{x})\mid y_{i})P(y_{i}), $$

where the conditional *p*(*f*_*i*_(**x**)∣*y*_*i*_) is the probability density of the feature vector for voxels with label *y*_*i*_ and *P*(*y*_*i*_) is the prior probability of a pixel having the label *y*_*i*_. We model the conditional distribution as a Gaussian,

10 $$ p(f_{i}(\mathbf{x})\mid y) \,=\, \frac{1}{\sqrt{(2\pi)^{k}\left|{\Sigma}_{y}\right|}} \exp\left( -\frac{1}{2}\left( f_{i}(\mathbf{x}) \,-\, \boldsymbol\mu_{y}\right)^{T}{\Sigma}_{y}^{-1}\left( f_{i}(\mathbf{x}) - \boldsymbol\mu_{y}\right)\right), $$

where the parameters ***μ***_*y*_ and Σ_*y*_ are the mean vector and the covariance matrix of the feature vectors for voxels with the label *y*, and *k* is the dimension of the feature vector.

In the training step these parameters are estimated from training data. The user manually labels a few voxels of the stack. During training, the voxels labeled with label *y* are used to estimate ***μ***_*y*_, i.e., the mean of the feature vectors of voxels labeled as *y*, and Σ_*y*_, i.e., the covariance matrix of these feature vectors.

When the dimension of the feature vectors is large, the training data often falls in a proper subspace of the complete *k*-dimensional feature space producing a singular or near singular covariance matrix Σ_*y*_. We avoid this problem by first performing Principal Component Analysis (PCA)-based dimensionality reduction on the cloud of all feature vectors . The dimensionality after the PCA is established to retain 99 % of variance.

*P*(*y*) is the a priori probability of the label *y*. It is learned from the user-provided data in the training step. In short, the training step consists of estimating the parameters ***μ***_*y*_ and Σ_*y*_ of the conditional distribution (after a PCA-based dimensionality reduction) and the prior *P*(*y*) from the user-provided data with the interactive tool.

**Fig. 3:**
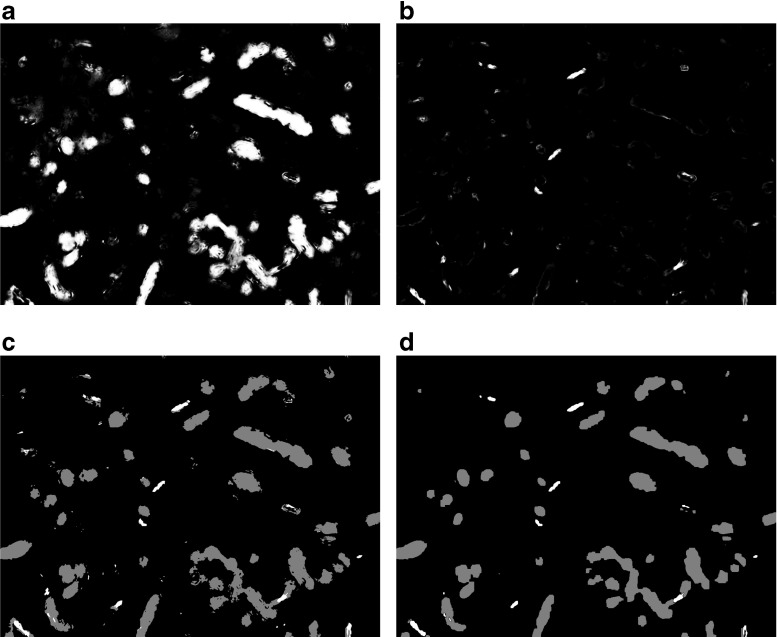
Classification and regularization of a single image. (**a**) and (**b**): Probability maps obtained by a Gaussian classifier that has been trained with the features extracted from the same image that is shown in Figure 2. The pixel-wise probability of belonging to the label ”Mitochondrion” is shown in (**a**) and the pixel-wise probability of belonging to the label ”Synaptic junction” is shown in (**b**). (**c**): preliminary segmentation before regularization, where each pixel has simply been given the label with highest probability (Mitochondria, gray; Synaptic junctions, white). Note the sparse pixels scattered throughout the image, the small holes in some of the segmented objects and the jagged edges. (**d**): Final segmentation after regularization via CRF energy minimization. Most sparse pixels have disappeared and edges show a smoother appearance

Once the classifier is trained, it processes every voxel in the EM stack. For each voxel *i* with feature vector *f*_*i*_(**x**), the probability *P*(*y*_*i*_∣*f*_*i*_(**x**)) is computed for every label *y*_*i*_. This results in a probability map that maps every voxel to the probabilities of belonging to each label. As an example, see Fig. 3a, b.

In preliminary experiments, we tested other classifiers such as support vector machines. Although these methods improve the results obtained with the Gaussian classifier, their performance is only marginally better at the expense of much higher computational time (in the order of hours vs. seconds), which makes them unsuitable for operation in real time.

#### Regularization

If voxels are assumed to be independent of each other, it is possible to segment the stack by simply assigning to each voxel *i* the label *y*^∗^ with higher probability, i.e., *y*^∗^ = *argmax*_*y*_*P*(*y*∣*x*). However, this offers far from optimal results, since the resulting segmentation is noisy, and it shows many sparse pixels, grainy regions and small holes (Fig. 3c, d).

Therefore, we have to assume some degree of probabilistic dependency between neighboring voxels of the stack. We have modeled this dependency by means of a conditional random field (CRF). A CRF models the distribution *P*(**Y**∣**x**) between the set of observed variables *x* (i.e., the pixel values of the stack of images) and the hidden variables **Y** = {*y*_1_,…, *y*_*N*_} (i.e., the segmentation labels) in such a way that **Y** conditioned on **x** holds the Markov property *P*(*y*_*i*_∣**x**, **Y**_−*i*_), where *Y*_−*i*_ is the set of label variables without *y*_*i*_, and *N*(*i*) are the neighboring voxels of voxel *i*. The neighborhood function *N* defines a graph (*V*, *E*) with the set of voxels *V* as nodes and the set of arcs *E* as given by the pair of voxels related by *N*. The graph constructed in this way is known as the CRF graph. The Hammersley–Clifford theorem states that the distribution of a CRF can be written as a Gibbs measure,

11 $$\begin{array}{@{}rcl@{}} P(\mathbf{Y}\mid\mathbf{x};\theta) &=& \frac{1}{Z(\mathbf{x},\theta)}\prod\limits_{c\in\mathcal{C}}{\Psi}_{c}(\mathbf{Y}_{c},\mathbf{x}_{c}) \\&=&\frac{1}{Z(\mathbf{x},\theta)}e^{-\sum\limits_{c\in\mathcal{C}}\theta_{c}\cdot \phi_{c}(\mathbf{Y}_{c},\mathbf{x}_{c})}, \end{array} $$

where *Z*(**x**, *θ*) is the partition function, $\mathcal {C}$ is the set of cliques in the CRF graph and **Y**_*c*_ is the set of variables from **Y** related to clique *c*.

In other words, the Gibbs measure expresses the distribution as the product of potentials Ψ_*c*_ (note that the potentials are not required to be probability distributions) depending on subsets of the complete set of random variables. The product of all potentials can be written as a weighted sum of factors using the minus logarithm,

12 $$ -\log \prod\limits_{c\in\mathcal{C}}\mathbf{\Psi}_{c}(\mathbf{Y}_{c},\mathbf{x}_{c}) = \sum\limits_{c\in\mathcal{C}}\theta_{c}\cdot \phi_{c}(\mathbf{Y}_{c},\mathbf{x}_{c}). $$

that, when written for a fixed observation **x**, is known as the energy of the labeling and denoted as *E*(**Y**). This energy is a map from the space of labelings to the real numbers. For improbable labelings the energy gives large values, whereas for good, probable labelings it provides lower values.

Finding the best segmentation with the probability distribution of the CRF, that models some degree of dependency among neighboring voxels, requires maximizing the probability *P*(**Y**∣**x**; *θ*) for a fixed observation **x**. This is equivalent to minimizing the energy *E*(**Y**).

**Fig. 4:**
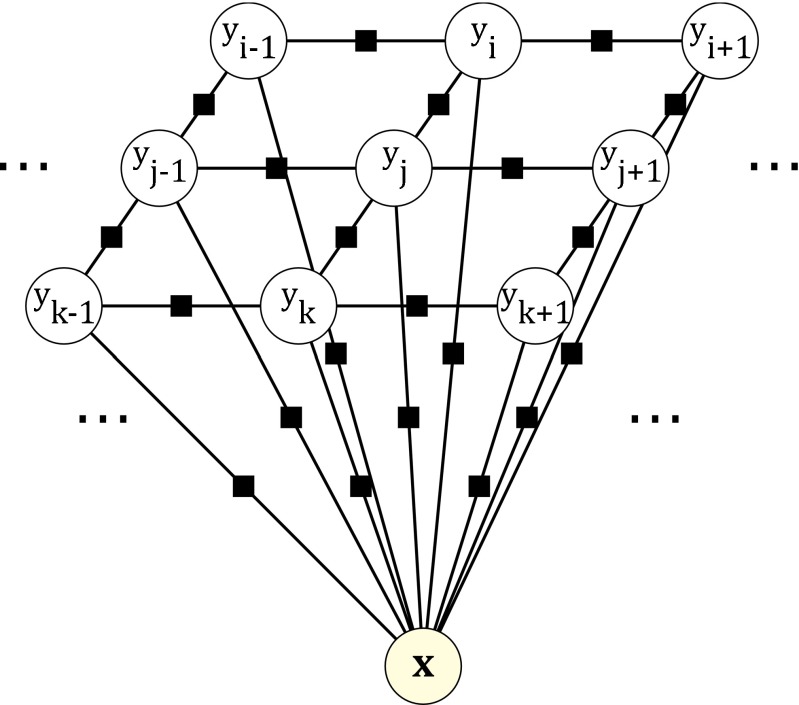
Factor graph for the CRF. The random variables are inside circle nodes. Black squares represent potentials depending on the random variables they are connected to. The energy of the CRF is the sum of all potentials. This figure shows only a fragment of a 2D CRF, but the generalization to 3D is straightforward

Figure 4 depicts the factor graph of our CRF. The factor graph is a bipartite graph that shows the random variables of the CRF graph as circles, as well as the potentials as little black squares. A potential depends on the random variables that it is connected to. Therefore, the given factor graph represents the following factorization of our distribution:

13 $$ P(\mathbf{Y}\mid\mathbf{x};\theta) \,=\, \frac{1}{Z(\mathbf{x},\theta)} \exp\left( -\sum\limits_{i\in V}\theta_{i}\cdot\phi_{i}(y_{i},\mathbf{x}) - \sum\limits_{(i,j)\in E}\theta_{ij}\cdot\phi_{ij}(y_{i},y_{j}) \right) $$

There are two kinds of potentials in this graph. The first kind of potential is associated with the terms *ϕ*_*i*_(*y*_*i*_, **x**). We will call them unary terms, since they only depend on a single variable *y*_*i*_ for a fixed observation in the energy function. The second kind of potential is related to the terms *ϕ*_*ij*_(*y*_*i*_, *y*_*j*_). In an analogous way, we will call them pair-wise terms, since they depend on pairs of label variables.

Training a CRF consists of determining its parameters *θ*. This tends to be a complex task, especially if the CRF has many parameters, as in our case. We therefore need to simplify it further. A very common and reasonable assumption is that the CRF is translation and orientation invariant, and as a consequence all of the parameters for a kind of term (unary or pairwise) share the same value. This would lead to the energy function:

14 $$ \theta_{1}\sum\limits_{i\in V}\phi_{i}(y_{i},\mathbf{x}) + \theta_{2}\sum\limits_{(i,j)\in E}\phi_{ij}(y_{i},y_{j}). $$

Unfortunately, in non-isotropic stacks we cannot assume orientation invariance. Usually, the stack has a lower resolution in the Z axis than in the X and Y axes. Therefore, we must treat the pair-wise terms that are oriented in the Z axis in a different way. We divide the set *E* into two disjoint subsets, *E*_*XY*_ and *E*_*Z*_, for the edges oriented along the X and the Y axes and for the edges oriented along the Z axis, respectively. The energy is now:

15 $$\begin{array}{@{}rcl@{}} \theta_{1}\sum\limits_{i\in V}\phi_{i}(y_{i},\mathbf{x}) &+& \theta_{XY}^{\prime}\sum\limits_{(i,j)\in E_{XY}}\phi_{ij}(y_{i},y_{j}) \\&+& \theta_{Z}^{\prime}\sum\limits_{(i,j)\in E_{Z}}\phi_{ij}(y_{i},y_{j}). \end{array} $$

Finally, since we are interested in the minimum energy, we can multiply the energy by $\frac {1}{\theta _{1}}$ and the solution remains unchanged:

16 $$\begin{array}{@{}rcl@{}} \sum\limits_{i\in V}\phi_{i}(y_{i},\mathbf{x}) &+& \theta_{XY}\sum\limits_{(i,j)\in E_{XY}}\phi_{ij}(y_{i},y_{j}) \\&+& \theta_{Z}\sum\limits_{(i,j)\in E_{Z}}\phi_{ij}(y_{i},y_{j}). \end{array} $$

This energy has only two parameters, *θ*_*XY*_ and *θ*_*Z*_, which control the strength of the regularization in the XY plane and in the Z axis. In an anisotropic stack we can assume that *θ*_*XY*_ and *θ*_*Z*_ are related by the expression $\theta _{Z}=\frac {\theta _{XY}}{\rho }$, and only one of them needs to be estimated by cross-validation or manually by the user. The manual estimation is further facilitated by the fact that the CRF offers good results for a large range of parameter values.

The unary and pair-wise terms have to be defined in such a way that they provide lower values for good, probable inputs. The unary terms *ϕ*_*i*_(*y*_*i*_, **x**) are responsible for introducing the observed data into the energy value. It is customary to define the unary terms as the minus logarithm of the probability that our trained classifier provides: *ϕ*_*i*_(*y*_*i*_, **x**) = −log*P*(*y*_*i*_∣*f*_*i*_(**x**)). This definition is justified since, in the absence of the pair-wise terms, the CRF would lead to the segmentation given by the classifier acting on each voxel separately.

The role of the pair-wise terms *ϕ*_*ij*_(*y*_*i*_, *y*_*j*_) is twofold. First, they regularize the segmentation results by penalizing the change of labels between neighboring voxels. This prevents the occurrence of isolated pixels and small holes that could appear (Fig. 3c, d). Second, they serve to introduce some extent of high-order knowledge about the structure of the stack. For example, we could impose the condition that synaptic junctions and mitochondria cannot touch each other, by setting a very large penalty to that label change in our experiments.

Therefore, the pair-wise terms are assigned as follows. A low penalty 1 is given to pairs of labels that are allowed to be neighbors. Second, a very high penalty *∞* is assigned to pairs of labels that cannot be adjacent (e.g., synaptic junctions and mitochondria). Third, no penalty is given for pairs of neighboring pixels with the same label. The distance matrix between labels *background* (bg), *synaptic junction* (syn) and *mitochondria* (mit) is therefore:

$$\begin{array}{@{}rcl@{}} &&\hspace*{2.5pc} bg\quad syn\quad mit\\ &&\begin{array}{l} bg\\ syn\\ mit\\ \end{array} \left( \begin{array}{lll} 0&~~~~~~1&~~~~~1\\ 1 & ~~~~~~0 &~~~~\infty\\ 1 & ~~~~~\infty & \quad~0\\ \end{array}\right). \end{array} $$

Once we have defined our energy, we need to find the segmentation that minimizes the energy function, **Y**^∗^ = *argmin*_**Y**_*E*(**Y**). This is in general an NP-hard optimization problem. However, it is known that when the terms are up to order two, i.e., there are only pair-wise (order 2) and unary (order 1) terms, the number of labels is two and the pair-wise terms are submodular, i.e., *ϕ*(*y*_*i*_, *y*_*i*_) + *ϕ*(*y*_*j*_, *y*_*j*_) ≤ *ϕ*(*y*_*i*_, *y*_*j*_) + *ϕ*(*y*_*j*_, *y*_*i*_), then a max-flow/min-cut algorithm finds the global optimum of the energy in polynomial time.

**Fig. 5:**
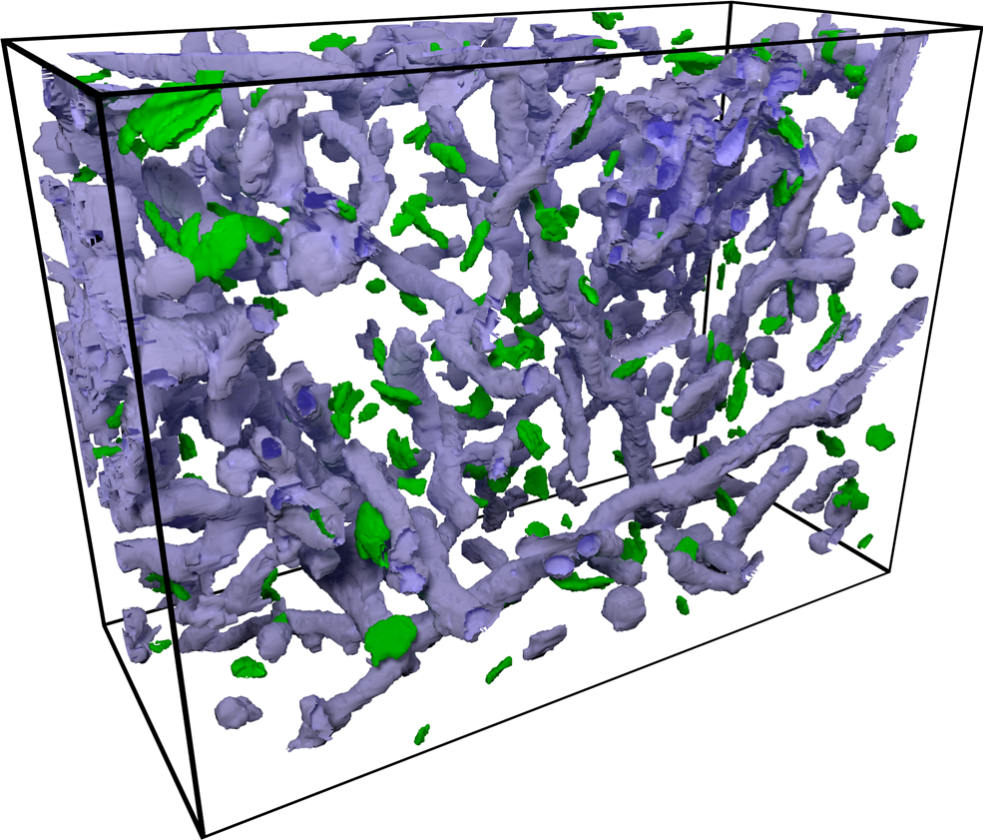
Segmentation of a whole stack of serial images after the regularization step. Mitochondria are shown in purple and synaptic junctions are shown in green

Unfortunately, when there are more than two labels, the max-flow algorithm is no longer applicable. Instead, we have to rely on approximate energy minimization using the *αβ*-swap algorithm from (Boykov et al. 2001). Figure 3c, d shows the segmentation of a single image after the *αβ*-swap regularization. Figure 5 shows the segmentation of a whole stack of serial EM images.

The graph-cut techniques needed for regularization require a considerable amount of computer memory. For a reasonably sized stack, the required memory usage usually becomes too big. Therefore we need to regularize parts of the full stack separately and merge them together at the end.

A simple approach is to divide the stack into disjoint, i.e., non-overlapping substacks and regularize them separately. This method works well for the inner areas of each substack, but it looks jumpy in their boundaries, since the CRF does not have enough context to determine the correct labels. This is visually noticeable as abrupt terminations of mitochondria and synaptic junctions at these boundaries.

**Fig. 6:**
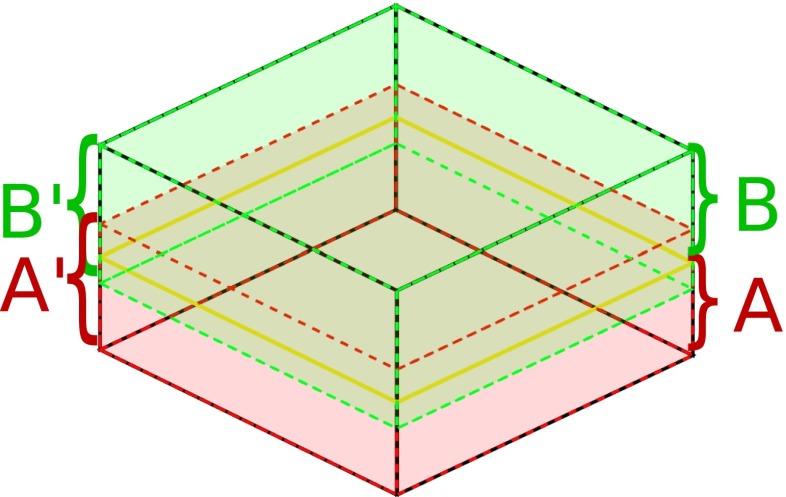
Regularization in overlapping partitions. Example of partition of a full stack into two disjoint substacks **A** and **B** and two overlapping substacks **A**’ and **B**’. Once the regularization has been performed in **A**’, only voxels belonging to **A** are updated. Then, regularization is performed in **B**’ and only voxels belonging to **B** are updated. This procedure prevents the appearance of regularization artifacts in the boundary between **A** and **B**

The solution to this problem consists of extending each substack with a margin, effectively making the new extended substacks overlap with their neighbors. The regularization is then applied to the extended substacks, but only the results obtained in the original substack volume are preserved in the final segmentation (Fig. 6).

Determining the optimal size of the margin is a problem beyond the scope of this paper. However, we have found that a margin of 10 voxels in each direction offers very good results in practice.

Finally, the size of the substacks is limited by the available memory. As a rule of thumb, the regularization process takes 5 or 6 times the memory used by the original substack being regularized.

#### Segmentation Smoothing

**Fig. 7:**
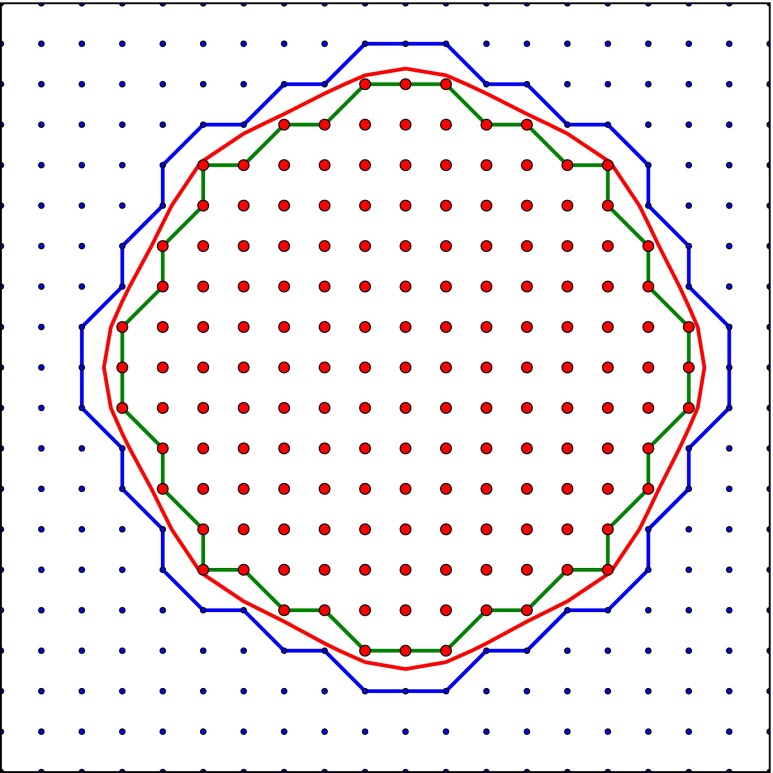
Example of a smoothed surface that meets the constraints imposed by the segmentation. The red and blue dots indicate the pixels that are inside and outside the object, respectively. The green and blue contours are the maximum and minimum area contours, respectively. Although there are infinite contours that would lie between them, we look for the smoothed minimum curvature contour depicted in red

The segmentation we obtain consists of hard label assignments to each voxel of the stack. This is suitable for several tasks such as counting of labeled objects or the estimation of the volume of the segmented objects, but presents disadvantages concerning the visualization of their surfaces. We use the marching cubes algorithm to extract the surfaces of the objects from the labels in the segmentation volume. This process not only produces unpleasant and unnatural renderings, but also biases the area estimates. Therefore we need to smooth the surfaces, but at the same time we want to preserve the constraints imposed by the labels in the segmentation volume. Among the infinite surfaces that meet these constraints, we compute the surface that minimizes curvature (see Fig. 7). To this end, we have adapted the method described by (Lempitsky 2010) to anisotropic stacks.

First, we build a vector of constraints $\{v_{i}\}_{i=1}^{N}\in \{-1,1\}^{N}$ such as *v*_*i*_ = 1 if the voxel *i* is inside an object and *v*_*i*_ = −1 if it is outside an object, i.e., if it has the background label. We find a real-valued vector $\{f_{i}\}_{i=1}^{N}\in \mathbb {R}^{N}$ such that *f*_*i*_ > 0 if *v*_*i*_ = 1 and *f*_*i*_<0 if *v*_*i*_ = −1 and its zero-levelset is smooth. In a continuous setting we would minimize the functional

17 $$ f^{*} = \arg\min_{f} {\int}_{\Omega} \left( \frac{\partial^{2} f}{\partial x^{2}}\right)^{2} + \left( \frac{\partial^{2} f}{\partial y^{2}}\right)^{2} + \left( \frac{\partial^{2} f}{\partial z^{2}}\right)^{2}\,\mathrm{d}V. $$

The minimization of the above functional smooths the segmentation result. In an anisotropic discrete setting, the smoothing problem becomes

18 $$\begin{array}{@{}rcl@{}} f^{*} &=& \min_{f} \sum\limits_{i} \left( f_{N_{x}(i)} + f_{N_{-x}(i)} - 2f_{i}\right)^{2} \\ &+& \left( f_{N_{y}(i)} + f_{N_{-y}(i)} - 2f_{i}\right)^{2} \\&+& \frac{\left( f_{N_{z}(i)} + f_{N_{-z}(i)} - 2f_{i}\right)^{2}}{\rho^{4}}, \end{array} $$

subject to

19 $$ v_{i}\cdot f_{i} \ge m_{i} \quad \forall i, $$

where *N*_[−]*d*_(*i*) is the neighbor of *i* in the direction (−)*d*, and $\{m_{i}\}_{i=1}^{N}$ is a vector of margins imposing the deviations from zero of *f* at each point. *m*_*i*_ is 0 if *i* is at the boundary of an object (i.e., if *v*_*i*_ and *v*_*j*_ for any of the neighbors *j* of *i* have different values) and 1 everywhere else. Here we have included factor *ρ* in the second derivative along the Z-axis to account for the anisotropy of the stack.

**Fig. 8:**
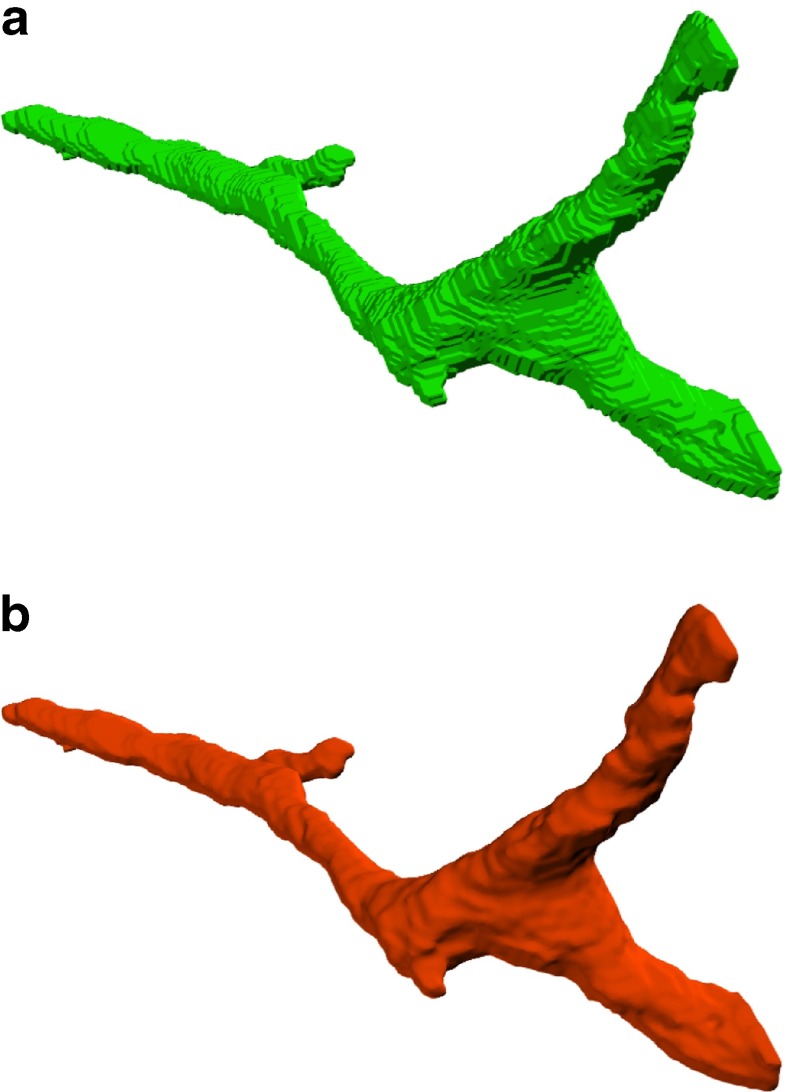
Example of a branching mitochondrion. A large branching mitochondrion is shown before (**a**) and after smoothing (**b**)

The Eqs. (18) and (19) constitute a convex quadratic program that can be solved with the Jacobi method with a slight modification to include the constraints in the algorithm (Lempitsky 2010). Figure 8 shows a mitochondrion before and after smoothing with this method.

Note that this smoothing only affects the estimated surfaces and, therefore, the rendering of these surfaces. The segmentation volume and the numerical results extracted from it are not affected by this procedure. Therefore, the quantitative comparisons offered in the Section “Results” are computed with no smoothing.

## Results

As explained in the previous section, we conceived our algorithm to be used interactively. However, to evaluate its performance we have used two datasets that have been fully segmented manually. We need these manual segmentations as ground-truth data to validate our results and compare them to others. Moreover, given that we have enough training data, we use them to find optimum values for the base scale *σ*_0_ and the regularization term *σ*_*XY*_.

We have used several voxel-based metrics to evaluate the quality of the segmentations. Voxel-based metrics measure the error rates in voxel classification taking into account true positive (TP), true negative (TN), false positive (FP) and false negative (FN) classifications.

- True positive rate (TPR):
  20 $$ \text{TPR} = \frac{\text{TP}}{\text{TP}+\text{FN}} $$
- False positive rate (FPR):
  21 $$ \text{FPR} = \frac{\text{FP}}{\text{FP} + \text{TN}} $$
- Accuracy (ACC):
  22 $$ \text{ACC} = \frac{\text{TP}+\text{TN}}{\text{TP}+\text{TN}+\text{FP}+\text{FN}} $$
- Jaccard index (JAC):
  23 $$ \text{JAC} = \frac{\text{TP}}{\text{TP} + \text{FP} + \text{FN}} $$
- Volume error (VOE):
  24 $$ \text{VOE} = \frac{\left|\text{FP} - \text{FN}\right|}{\text{TP} + \text{FN}} $$

Unless otherwise stated, all running times were obtained on a Linux system with an Intel Xeon at 2.40GHz with no GPU processing. Our algorithm is mostly implemented in Python using the NumPy library. The inner parts of the CRF regularization are written in C++. Our implementation runs in a single thread.

### Mitochondria Segmentation

We used a stack of serial EM images from the mouse cerebellum to test our method for the segmentation of mitochondria. This stack is available online in the Cell Centered Database^1^ with ID 8192. We have selected this stack to make our method comparable with Cytoseg, an automatic segmentation tool proposed by (Giuly et al. 2012). These researchers provide the raw images as well as a manual segmentation of mitochondria at the Cytoseg web page.^2^ The stack has a size of 700×700×50 voxels and a voxel size of 10×10×50 nm (anisotropy factor *ρ* = 5). We have applied our method to automatically detect the mitochondria in this stack.

**Fig. 9:**
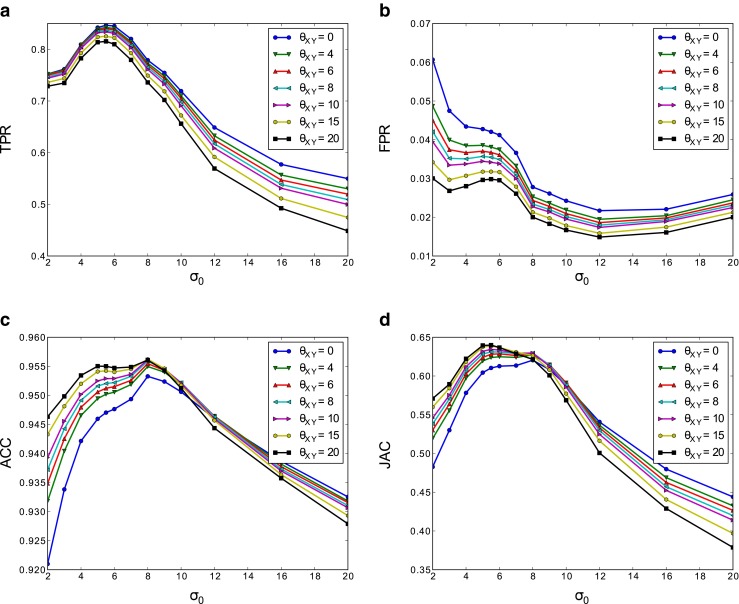
Metrics obtained by cross-validation for several values of the parameters *σ*_0_ and *θ*_*XY*_. (**a**) TPR vs. *σ*_0_ for different values of *θ*_*XY*_. (**b**) FPR, (**c**) ACC and (**d**) JAC

**Fig. 10:**
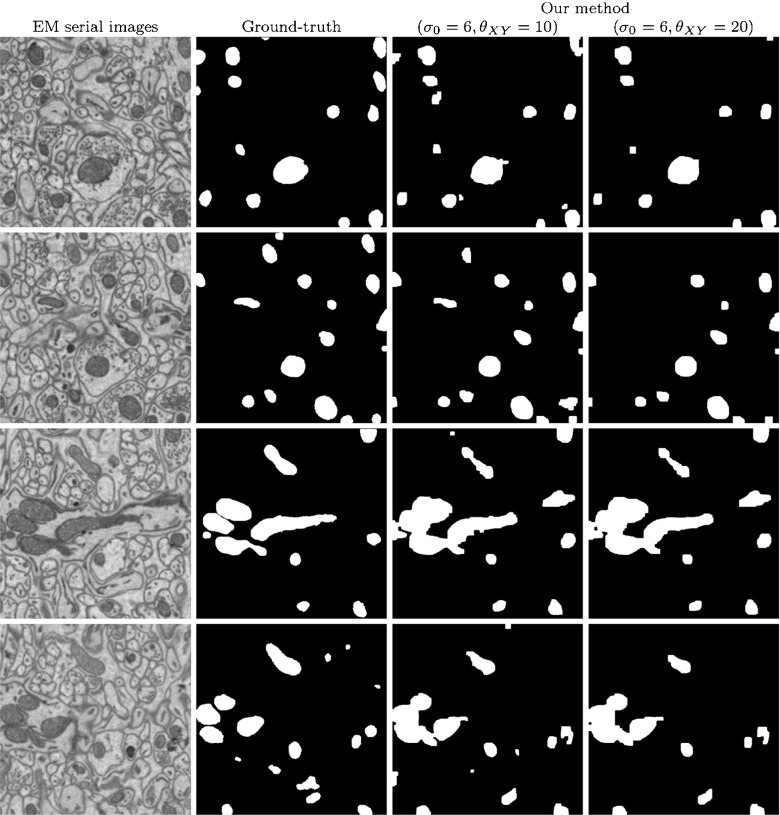
Segmentation of mitochondria in a 700×700×50 stack of EM serial images. The left column shows four individual, non-consecutive images from the stack. The second column shows ground truth data, manually segmented by an expert. The two rightmost columns show the results obtained with our algorithm using a base scale *σ*_0_ = 6 and two different sets of regularization parameters *θ*_*XY*_ and *θ*_*Z*_.

The stack was divided into 5 sets of 10 images and we used 5-fold cross-validation to estimate the quality of the segmentation for different pairs of values of *σ*_0_ and *θ*_*XY*_. Values of *σ*_0_ ranged from 2 to 20 and values of *θ*_*XY*_ ranged from 0 to 20. Figure 9 plots the TPR, FPR, ACC and JAC metrics obtained. The curves show that the base scale for feature extraction *σ*_0_ is much more critical for the quality of the segmentations than the regularization penalty *θ*_*XY*_. In fact, the regularization penalty is almost irrelevant for the quantitative measurements, but it is much more important in the visual or qualitative results (see Fig. 10).

From the results of the cross-validation process, we choose *σ*_0_ = 6 as it offers a good trade-off between the considered metrics. For the regularization parameter *θ*_*XY*_, we select two different values: 10 and 20. The parameter $\theta _{Z}=\frac {\theta _{XY}}{\rho }$ is set to 2 and 4, respectively.

After choosing the parameters, we apply our segmentation algorithm to the full stack. We used the last 10 slices of the stack for training. The results obtained with our method are similar to those obtained with the Cytoseg process (see Table 1). However, Cytoseg (Giuly et al. 2012) required 80 minutes of processing time for a stack of 350×350×30 according to their paper, while our algorithm took 53.8 seconds for the segmentation of a 700×700×50 stack (training takes 9.6 seconds and the complete stack labeling, including CRF regularization, 44.2 seconds).

**Table 1:** Comparative results for mitochondria detection performed by our algorithm with two different sets of regularization parameters, by Ilastik (Sommer et al. 2011), and by the Cytoseg process, according to (Giuly et al. 2012)

Method	TPR	FPR	ACC	JAC	VOE
Ours, *σ*_0_ = 6; *θ*_*XY*_ = 10; *θ*_*Z*_ = 2	0.78	0.018	0.96	0.68	8.26 %
Ours, *σ*_0_ = 6; *θ*_*XY*_ = 20; *θ*_*Z*_ = 4	0.81	0.024	0.96	0.67	2.51 %
Cytoseg	0.80	0.02	0.97	N/A	N/A
Ilastik	0.77	0.02	0.96	0.66	5.45 %

We also applied the software Ilastik ((Sommer et al. 2011), www.ilastik.org) to segment mitochondria in this dataset. The quantitative results obtained in Ilastik (see Table 1) are comparable to those of the other methods. However, Ilastik took 56.5 minutes for processing the full stack using 8 threads, resulting in a total of 452 minutes of CPU. This is about 500 times slower than our method.

Other methods for mitochondria segmentation are even less suitable for large anisotropies. Supervoxel segmentation with learned shape features (Lucchi et al. 2012) aims to learn non-local shapes of the target objects to segment. They use a combination of supervoxels, 3D ray features and structured prediction. 3D ray features are specially affected by anisotropy since both the edge detector and the length of the rays are highly dependent on the orientation. The achievable segmentation accuracy —i.e., the highest accuracy that can be achieved using supervoxels assuming perfect classification of each supervoxel— drops significantly with anisotropy. Moreover, the structured prediction requires training with a large portion of the stack fully labeled in order to infer the terms of the pairwise interaction. As a consequence of these factors, the method from (Lucchi et al. 2012) required more training data (half of the stack) to work properly, and provided rather unsatisfactory results with low Jaccard indices (<0.48). The running times were also higher (>21 minutes) due mainly to the cost of extraction of the ray features.

### Mitochondria and Synaptic Junctions Segmentation

**Fig. 11:**
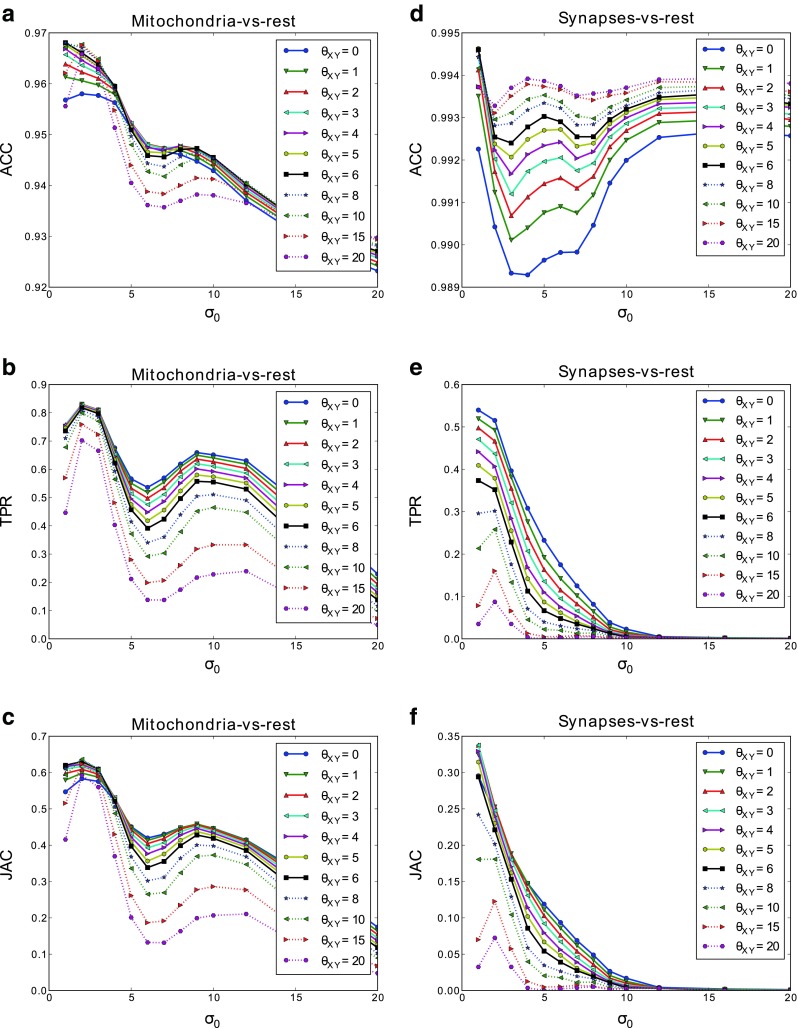
Simultaneous segmentation of mitochondria and synaptic junctions. Metrics obtained by cross-validation for several values of the parameters *σ*_0_ and *θ*_*XY*_. ACC, TPR, and JAC are shown for mitochondria (**a**–**c**) and synaptic junctions (**d**–**f**)

For this test we used a stack of 366×494×213 voxels and a voxel size of 14.7×14.7×20 nm (*ρ* = 1.36) acquired from the rat somatosensory cortex. We used the first 100 slices of the stack to estimate the parameters of the algorithm with 5-fold cross-validation. The results of the cross-validation are plotted in Fig. 11. Again, the base scale *σ*_0_ had a critical impact on performance, whereas the regularization penalty *θ*_*XY*_ only caused subtle variations. Note that we were segmenting three different classes (background, mitochondria and synapses) and therefore we measured the quality of segmentations with mitochondria-vs-rest and synapses-vs-rest metrics, i.e., considering one of the classes as foreground and the rest as background.

**Fig. 12:**
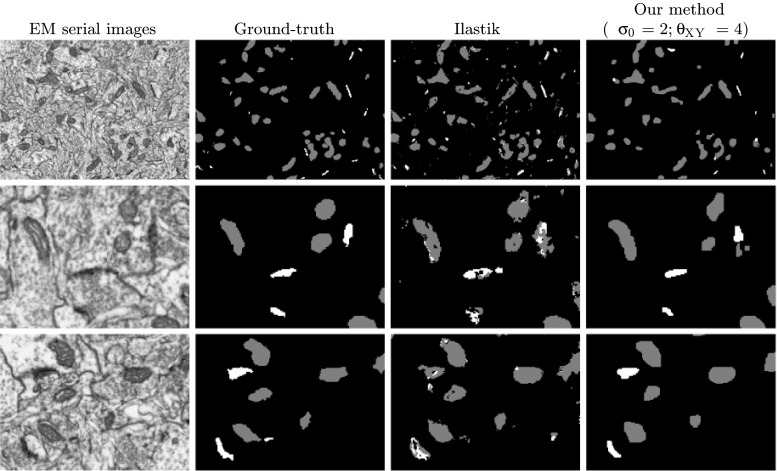
Simultaneous segmentation of mitochondria and synaptic junctions. The left column shows the original images; the center left column is the ground-truth; the center right column is the result obtained with Ilastik; the right column is the result of our algorithm with parameters *σ*_0_ = 2; *θ*_*XY*_ = 4; *θ*_*Z*_ = 2.94. The first row shows a full slice. The second and third rows show zoomed regions of different slices for detail

From the results of cross-validation, we chose *σ*_0_ = 1 and *σ*_0_ = 2 as a trade-off value that worked reasonably well for both mitochondria and synaptic junctions. We set *θ*_*XY*_ = 4 and $\theta _{Z} = \frac {\theta _{XY}}{\rho } = 2.94$. The training was performed using 11 evenly distributed slices of the stack and it took 3.27 seconds to complete. The segmentation of the full stack (213 serial images) took 10.15 minutes (48.31 seconds for the classification step and the rest for regularization). Figure 12 shows the results of our algorithm and Ilastik. Table 2 compares the quantitative performance of both algorithms. The Ilastik performance results were obtained using a very similar manual segmentation to the one used with our algorithm. The results obtained with both methods were similar when considering the numerical performance, with ours being marginally better. However, Ilastik took 56 minutes with 8 threads to train and segment the full stack, making our method 45 times faster. Visual appearance of the final segments were also much better in our case thanks to the regularization procedure (see Fig. 12).

**Table 2:** Quantitative results for the simultaneous segmentation of mitochondria and synaptic junctions

Method	TPR	FPR	ACC	JAC	VOE
Ours, mit-vs-rest, *σ*_0_ = 1, *θ*_*XZ*_ = 4, *θ*_*Z*_ = 2.94	0.84	0.02	0.97	0.60	15.72 %
Ours, syn-vs-rest, *σ*_0_ = 1, *θ*_*XZ*_ = 4, *θ*_*Z*_ = 2.94	0.60	0.004	0.99	0.26	87.17 %
Ours, mit-vs-rest, *σ*_0_ = 2, *θ*_*XZ*_ = 4, *θ*_*Z*_ = 2.94	0.78	0.014	0.97	0.63	0.01 %
Ours, syn-vs-rest, *σ*_0_ = 2, *θ*_*XZ*_ = 4, *θ*_*Z*_ = 2.94	0.42	0.004	0.99	0.23	24.00 %
Ilastik, mit-vs-rest	0.68	0.009	0.97	0.61	21.58 %
Ilastik, syn-vs-rest	0.36	0.002	0.99	0.29	41.43 %

### Running Time Comparison

Table 3 summarizes running times for our experiments in both datasets. Our method runs much faster compared to the others with similar or better performance. Cytoseg and learned shape features are specialized in mitochondria segmentation; thus, we only report results in the first dataset for those methods.

**Table 3:** Absolute and normalized running times for different methods. Absolute times are given in seconds of CPU (s ⋅CPU), and normalized times (in parentheses) are given in seconds of CPU per megavoxel $\left (\frac {\mathrm {s}\cdot \textrm {CPU}}{\text {Megavoxel}}\right )$

Method	CCDB-8192	Mit&Syn
Cytoseg (Giuly et al. 2012)	4800 (192.08)	N/A
Ilastik (Sommer et al. 2011)	14216 (568.87)	27112 (704)
Learned shape features (Lucchi et al. 2012)	1380 (55.22)	N/A
Ours	53.8 (2.15)	612.27 (15.9)

There is an important difference in running times for our method in both datasets (2.15 vs. 15.9). This large difference is due to the regularization with >2 labels, where a single graph-cut is inviable and iterative, slower algorithms such as *αβ*-swap are required.

### Counting Structures

Estimating the number of structures from the results of an automatic segmentation process is still an open problem with plenty of ongoing research. As an approximate, simple solution, it is commonly assumed that each connected component of the segmentation is one structure. This is the approach we use. Despite its simplicity, it has several drawbacks, namely, a group of structures close to each other often merge in a single connected component, and large structures are sometimes split into two or more connected components. Also, when spatial regularization is not present, false positive detections result in many small connected components that bias the counting estimations. To alleviate these problems, we discard the connected components smaller than a given threshold. Setting the threshold is not trivial, as it might greatly affect the counts depending on the quality of the segmentation. A good segmentation is expected to be more robust to different thresholds than a bad one, i.e., estimations should be close to the real value and should be stable for a large range of thresholds.

**Fig. 13:**
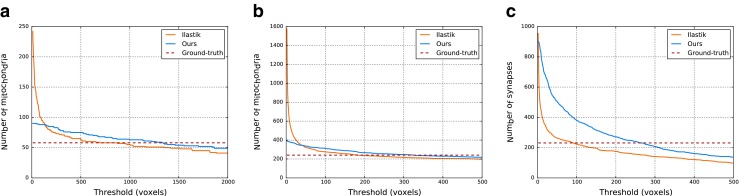
Count estimations for varying thresholds. (**a**) shows estimations in the number of mitochondria for the CCDB-8192 dataset. (**b**) and (**c**) show estimations in the number of mitochondria and synaptic junctions respectively in the Mit&Syn dataset

Figure 13 shows count estimations for different thresholds with both Ilastik and our method. The regularization makes our method more robust: it reduces the number of small components and the estimations of our method are closer to the ground-truth for a wider range of thresholds. Table 4 gives numerical assessment of this idea. It shows the absolute value of the deviations of the estimations from the ground-truth averaged over all thresholds in the range $T = [10, 2000]\subset \mathbb {Z}$:

25 $$ \frac{1}{|T|}\sum\limits_{t\in T} \left|\textrm{\#CC}_{[\text{size} \ge t]} - \text{GT}\right|, $$

where #CC_[size ≥ *t*]_ is the number of connected components with size ≥ *t*, and GT is the real count. Table 4 shows that our method has smaller errors than Ilastik for all datasets and considered structures.

**Table 4:** Average absolute error of estimations of the number of mitochondria and synaptic junctions over all thresholds in the range [10,2000] voxels

Method	CCDB-8192	Mit&Syn, Mit	Mit&Syn, Syn
Ilastik (Sommer et al. 2011)	12.12	68.44	166.17
Ours	**10.71**	**54.97**	**161.76**

## Discussion

Concerning the segmentation of mitochondria, Lucchi and colleagues (Lucchi et al. 2012) have recently used ray descriptors and the gray-level histogram as the key features to classify 3D image supervoxels. The result of this classification is further regularized using graph cuts to minimize an energy function involving learned potentials. They used stacks of FIB/SEM images from the hippocampus and striatum that had isotropic resolution. In their method, isotropy is an essential requirement for the computation of the 3D supervoxel over-segmentation. Alternatively, Giuly and colleagues (Giuly et al. 2012) segment mitochondria in anisotropic stacks of images obtained by SBFSEM. They use a random forest classifier to label 2D image patches. The result of this initial segmentation is further refined using 2D contour classification across images and 3D level-set surface evolution. Their method, however, is computationally intensive, requiring long processing times

Regarding synapses, the popular Ilastik toolbox (Sommer et al. 2011) used by (Kreshuk et al. 2011) to segment synaptic junctions uses a random forest classifier with a set of differential image features. They use a simple regularization strategy based on Gaussian smoothing. Overall, the resulting algorithm is also very demanding in terms of computing power.

Our method does not require isotropic voxels so it can be applied to image stacks that have been acquired with different resolution in the X, Y and Z axes. The results obtained with our method were similar or better than those obtained with the Cytoseg process (Giuly et al. 2012) for mitochondria only, and to those obtained with Ilastik for both mitochondria only and simultaneous mitochondria and synaptic junctions. Other approaches such as the one from (Lucchi et al. 2012) are not ready to work with anisotropic stacks and therefore our method outperforms them. Unlike Cytoseg, that focuses on mitochondria segmentation, our method is not tied to a specific type of cellular structure but can be used to segment a variety of structures. When compared to Ilastik we obtained better visual results thanks to the regularization and surface smoothing techniques described above.

Moreover, our method is much faster than any other approach we have tried. The speed up comes from the Gaussian classifier, that can be trained in *O*(*Nk*^2^ + *k*^3^), being *N* the number of data points and *k* the dimension of the feature space. For comparison, the complexity of training random forests is *O*(*MNkd*), being *M* the number of trees and *d* the average depth of the trees. We found in our experiments that the classifier was the main bottleneck of the Ilastik approach. In our approach the most expensive computation was the regularization step, which Ilastik omits. On the other hand, we found no significant difference in speed for feature extraction, taking only a small fraction of the total processing time in all compared methods.

For the case of segmentation of 2 labels, a speed of 2.15 seconds per megavoxel in a single thread is fast enough to enable interactive segmentation of the large image stacks that are now available, providing real-time feedback to the user. Of course, parallelization of the proposed approach is straightforward, and it would make it even faster. To our knowledge, no other previous work provides state-of-the-art performance while running in an interactive setting.

## Conclusions

We have presented an algorithm that can be trained to segment a variety of structures in anisotropic EM stacks. In this work we have focused on its capabilities for the segmentation of synaptic junctions and mitochondria. It features some important properties that are not available in other methods in the literature. It uses a graph cut-based image regularization procedure that not only provides better segmentations, but also introduces high level knowledge about the structure of labels. We have solved the limitation of graph cuts in terms of memory requirements with the introduction of energy optimization in overlapping partitions. This allows the regularization of very large stacks. The surface smoothing step introduces smoothness priors on the segmentation that improves the appearance of three-dimensional renderings of the segmented volumes. Finally, and most importantly, we have also shown that our approach is much faster than any other competing method with a state-of-the-art quantitative segmentation performance.

## Information Sharing Statement

The automatic segmentation method described in this paper is available as a plugin of the imaging processing software Espina. The software and instructions for installing it can be found at http://cajalbbp.cesvima.upm.es/espina.

This software provides an efficient multi-thread implementation of the presented algorithm together with an intuitive user interface. After activating the Automatic Segmentation plugin, the user has to segment a few voxels of the target objects manually and receives almost real-time feedback of the results. Additional manual segmentations can be performed until the user is satisfied with the final results. Quantitative data regarding the segmented objects are then obtained with standard Espina tools.

## Electronic supplementary material

Below is the link to the electronic supplementary material.

(AVI 21.8 MB)

## Footnotes

1 ^1^http://ccdb.ucsd.edu

2 ^2^https://code.google.com/p/cytoseg/
